# Supplementary material for: Prediabetes Is Independently Associated with Subclinical Carotid Atherosclerosis: An Observational Study in a Non-Urban Mediterranean Population
Source: J Clin Med. 2020 Jul 7;9(7):2139. doi: 10.3390/jcm9072139 (PMC7408832; doi:10.3390/jcm9072139)
Supplement: Supplementary file 1 [file jcm-09-02139-s001.pdf]

Table S1. Univariate analysis of for the presence of plaque presence in the whole study group.

	No plaque	Plaque	P-value
Sample size, N (%)	401 (72.9%)	149 (27.1%)	
Sex (Women)	254 (63.3%)	67 (45%)	<0.001
Age (years)	46.9 ± 12	58,6 ± 11.8	<0.001
Waist (cm)	91.8 ± 11.9	99.1 ± 11.4	<0.001
Tobacco exposure (%)	198 (49.4%)	85 (57%)	0.110
Total cholesterol (mg/dL)	197.4 ± 36	210.8 ± 34.9	<0.001
HDL-cholesterol (mg/dL)	59.4 ± 14.5	56.9 ± 15	0.033
LDL-cholesterol (mg/dL)	118.5 ± 30.2	131.2 ± 30.2	<0.001
Triglycerides (mg/dL)	99.9 ± 54	126.8 ± 125.9	0.001
eGFR (ml/min)	96.3 ± 14.7	88.6 ± 14.9	<0.001
Urate Serum (mg/dL)	4.7 ± 1.2	5.3 ± 1.3	<0.001
Leukocytes (x10x9/L)	6.4 ± 1.6	7 ± 1.9	0.003
Systolic blood pressure (mmHg)	118.9 ± 16.3	130.1 ± 15.1	<0.001
Diastolic blood pressure (mmHg)	75.6 ± 9.8	79.9 ± 10.3	<0.001

HDL, high-density lipoprotein LDL, low-density lipoprotein; eGFR, estimated glomerular filtration rate.
